# The Efficacy and Safety of Knotless Barbed Sutures in the Surgical Field: A Systematic Review and Meta-analysis of Randomized Controlled Trials

**DOI:** 10.1038/srep23425

**Published:** 2016-03-23

**Authors:** Yifei Lin, Sike Lai, Jin Huang, Liang Du

**Affiliations:** 1 Institute of Urology, Department of Urology, West China Hospital, Sichuan University, Guoxuexiang 37, Chengdu, Sichuan, 610041, China; 2 West China School of Medicine, Sichuan University, Guoxuexiang 37, Chengdu, Sichuan, 610041, China; 3 West China Hospital, Sichuan University, Guoxuexiang 37, Chengdu, Sichuan, 610041, China

## Abstract

The knotless barbed suture is an innovative type of suture that can accelerate the placement of sutures and eliminate knot tying. However, the outcomes of previous studies are still confounding. This study reviewed the application of different types of barbed sutures in different surgeries. We searched PubMed, EMBASE, CENTRAL and ClinicalTrials.gov to identify randomized controlled trials (RCTs) addressing the application of barbed sutures up to Feb. 2015. Two reviewers independently screened the literature and assessed the risk of bias of included studies. Then meta-analysis was performed using RevMan 5.3 software. Sensitivity analysis and subgroup analysis was performed. Seventeen RCTs (low to moderate risk of bias) involving 1992 patients were included. Compared with conventional sutures, the barbed suture could reduce suture time (SMD=−0.95, 95%CI −1.43 to −0.46, P = 0.0001) and the operative time (SMD=−0.28, 95%CI −0.46 to −0.10, P = 0.003), not significantly increase the estimated blood loss (SMD=−0.09, 95%CI −0.52 to 0.35, P = 0.70), but could lead to more postoperative complications (OR = 1.43, 95%CI 1.05 to 1.96, P = 0.03), These results varied in subgroups. Thus, barbed sutures are effective in reducing the suture and operative time, but the safety evidences are still not sufficient. It need be evaluated based on special surgeries and suture types before put into clinical practice.

The knotless barbed suture is a relatively new type of suture that has been widely used in both skin and deeper structures. It is a specifically designed monofilament suture with barbs orientated in the opposite direction to the needle. Generally, complications of conventional knot tying are well recognized; conventional knot tying requires time and training, and the knots may easily break or extrude. Infection related to knots is also frequently observed^1^. By contrast, the novel barbs on the ligatures make the suture grab the tissue, without allowing the suture to slide back.

Since their invention in 1964^2^, barbed sutures have now been applied in various fields, including cosmetic, urological, general, orthopedic, obstetric, gynecological, and other surgeries. Specifically, barbed sutures are available in both absorbable and non-absorbable monofilament materials. Currently, three types of barbed sutures^3^ are commercially available: the Quill SRS (Quill Self-Retaining System; Angiotech Pharmaceuticals, Vancouver, British Columbia, Canada), which is a bidirectional barbed suture; the V-Loc Absorbable Wound Closure device (Covidien, Mansfield, MA, USA), which is a unidirectional barbed suture that has only 1 needle and a loop at the end; and the Stratafix (STRATAFIX Knotless Tissue Control Devices, Ethicon Inc., Somerville, NJ, USA), which presents a spiral distribution of the barbs and anchors.

Although an increasing number of studies have reported the advantages of this technique, the outcomes of previous clinical trials are still confounding, and no studies have comprehensively examined the benefits. Thus, we present the available evidence in terms of the efficacy and safety of different types of knotless barbed sutures in different surgeries by performing a systematic review and meta-analysis of the current literature.

## Results

### Study selection process and characteristics

**Figure 1:**
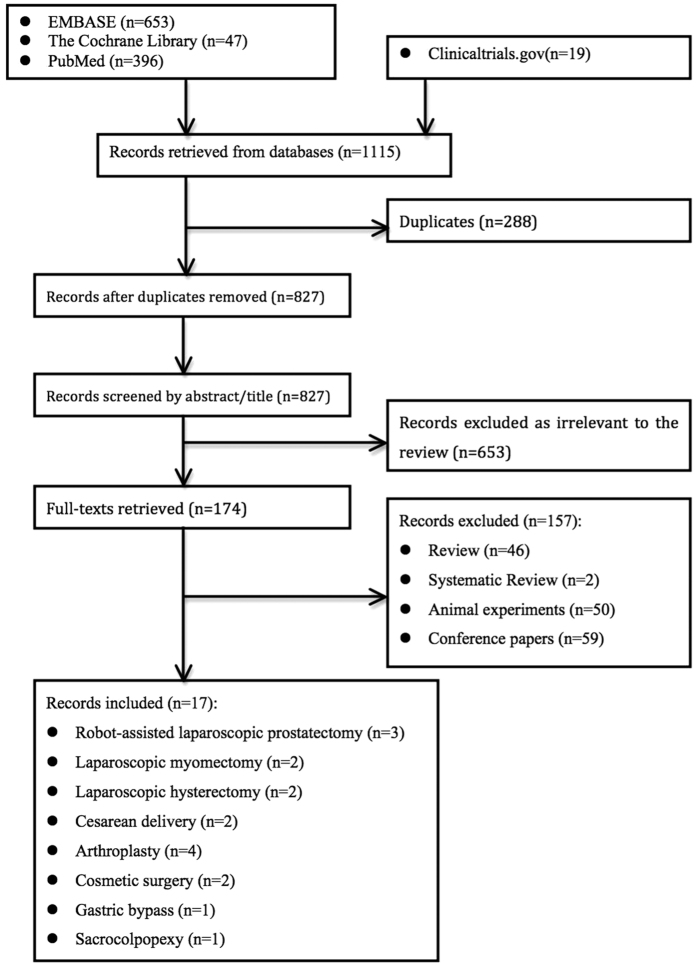
Flow diagram of the detailed selection process.

A total of 1115 records were identified after an initial search of selected electronic databases. A flow diagram of the detailed selection process is shown in Fig. 1. Finally, 17 randomized controlled trials (RCTs) involving 1992 surgical patients were included for further meta-analyses^4^,^5^,^6^,^7^,^8^,^9^,^10^,^11^,^12^,^13^,^14^,^15^,^16^,^17^,^18^,^19^,^20^. Among these studies, 3 were related to robot-assisted laparoscopic prostatectomy^7^,^8^,^10^, 2 were related to laparoscopic myomectomy^5^,^11^, 2 were related to laparoscopic hysterectomy^12^,^13^, 2 were related to cesarean delivery^4^,^6^, 4 were related to arthroplasty^9^,^16^,^18^,^20^, 2 were related to cosmetic surgery^14^,^17^, 1 was related to gastric bypass^15^, and 1 was related to sacrocolpopexy^19^. Table 1 provides the baseline characteristics of all studies.

Of the 17 trials, 16 trials were performed using computer-generated randomization, 1 used the coin toss; 9 performed allocation concealment through central randomization; 5 applied blinding only to patients and 1 was open labeled; and 4 applied blinding to outcome assessors while 1 did not. The loss to follow-up occurred in 0 to 14.1% of patients. In general, the risk of bias was low to moderate in RCTs (Supplementary Table 1).

### Quantitative data synthesis

The heterogeneity of barbed suture vs. conventional suture for all 17 studies was individually assessed and focused on different outcomes. Subgroup analyses were performed using different types of surgeries and barbed suture types (Table 2, Supplementary Figures 1–8).

### Suture time

**Figure 2:**
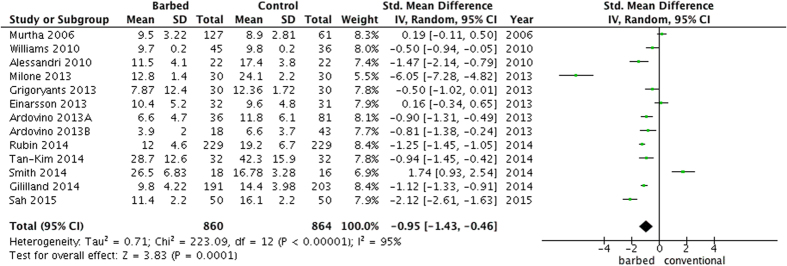
A forest plot of suturing time with or without barbed suture.

With regard to the suture time (Fig. 2), a barbed suture could significantly reduce the suture time (SMD =−0.95, 95%CI −1.43 to −0.46, P = 0.0001), but the heterogeneity was high (P < 0.00001, I^2^ =95%) among 8 surgeries^4^,^5^,^7^,^11^,^12^,^13^,^14^,^15^,^16^,^17^,^18^,^19^,^20^. In the subgroup analysis by different surgeries, a shorter suture time in the barbed suture group was observed in laparoscopic myomectomy (MD =−5.50, 95%CI −7.03 to −3.97, P < 0.0001), cosmetic surgery (MD =−6.76, 95%CI −8.72 to −4.79, P < 0.00001), sacrocolpopexy (MD =−13.60, 95%CI −20.63 to −6.57, P = 0.0001), gastric bypass (MD =−11.30, 95%CI −12.23 to −10.37, P < 0.00001) and robot-assisted laparoscopic prostatectomy (MD =−0.10, 95%CI −0.19 to −0.01, P = 0.03). In the subgroup analysis by different types of barbed suture, a significantly decreased suture time (SMD =−1.75, 95%CI −2.69 to −0.81, P = 0.0003) was found in the unidirectional barbed suture groups.

#### Operative time

**Figure 3:**
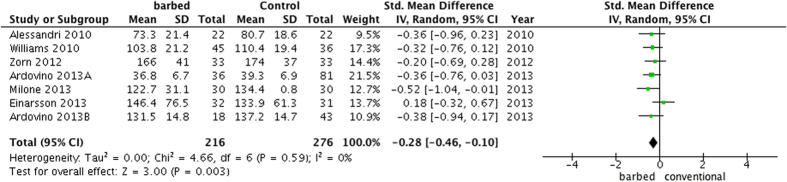
A forest plot of operative time with or without barbed suture.

In general, the operative time was significantly shorter (SMD =−0.28, 95%CI −0.46 to −0.10, P = 0.003) in the barbed suture group^5^,^7^,^10^,^11^,^12^,^13^,^15^ with lower heterogeneity (P = 0.59, I^2^ = 0%) (Fig. 3). In the subgroup analysis by different surgeries, a significantly shorter operative time in the barbed suture group was found in laparoscopic myomectomies (MD =−2.73, 95%CI −5.32 to −0.14, P = 0.04) and gastric bypass (MD = −11.70, 95%CI −22.83 to −0.57, P = 0.04). In the subgroup analysis by different types of barbed suture, a significant decreased operative time (SMD =−0.34, 95%CI −0.59 to −0.09, P = 0.001) was found in the unidirectional barbed suture groups.

#### Estimated blood loss

**Figure 4:**
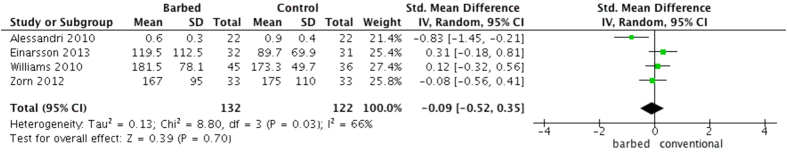
A forest plot of estimated blood loss with or without barbed suture.

The estimated blood loss (Fig. 4) changed insignificantly (SMD =−0.09, 95%CI −0.52 to 0.35, P = 0.70) with high heterogeneity (P = 0.03, I^2^ = 66%)^5^,^7^,^10^,^13^. In the subgroup analysis by different surgeries, estimated blood loss was significantly less in the barbed suture group only when referring to laparoscopic myomectomies (SMD =−0.83, 95%CI −1.45 to −0.21, P = 0.008). In the subgroup analysis by different types of barbed suture, no significant results were observed.

#### Postoperative complications

**Figure 5:**
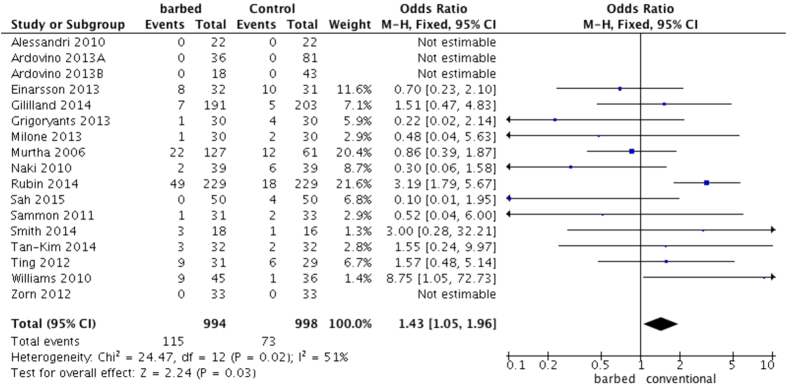
A forest plot of postoperative complications with or without barbed suture.

According to the pooled data, postoperative complications occurred more often in the barbed suture group than in the control group (OR = 1.43, 95%CI 1.05 to 1.96, P = 0.03)^4^,^5^,^6^,^7^,^8^,^9^,^10^,^11^,^12^,^13^,^14^,^15^,^16^,^17^,^18^,^19^,^20^. (Heterogeneity: P  = 0.02, I^2^  = 51%, Fig. 5) In the subgroup analysis by different surgeries, only cosmetic surgery appeared to significantly have more postoperative complications in the barbed suture group (SMD = 2.47, 95%CI 1.50 to 4.06, P = 0.0004). Rubin *et al*.^17^ suggested that suture extrusion was among the most common complications arising from mastopexy procedures (one of the cosmetic surgeries). In the subgroup analysis by different types of barbed suture, the unidirectional barbed suture groups had significantly more postoperative complications (OR = 2.13, 95%CI 1.35 to 3.35, P = 0.005). Because research performed by Rubin *et al*.^17^ involved more than one type of cosmetic surgery (abdominoplasty, mastopexy, and reduction mammoplasty) and William *et al*.^7^ had modified their technique for anastomosis of the bladder and urethral stump midway through the trials, we considered that these studies demonstrated more confounding variables. Moreover, a sensitivity analysis excluding these two studies showed no statistical change in postoperative complications between the conventional and unidirectional barbed sutures (OR = 0.30, 95%CI 0.09 to 0.98, P = 0.05, Supplementary Figure 9).

### Publication bias

Publication bias was assessed using Begg’s funnel plots. The shape of the funnel plots appeared symmetric in the barbed vs. conventional suture, suggesting no evidence of publication bias (Supplementary Figures 10–13).

## Discussion

Generally, barbed sutures reduced the suture time in nearly all types of surgeries, as well as the operative time. Although barbed sutures resulted in more postoperative complications, no significant change occurred concerning the estimated blood loss. Moreover, the results differed in different surgeries, and the bidirectional barbed suture appeared to be better than the unidirectional barbed suture.

To eliminate interference from confounding factors, we performed subgroup analysis by surgeries and barbed type, and the results were varied. First, our subgroup results showed a significant association between suture time and barbed suture in 5 types of surgeries (laparoscopic myomectomies, cosmetic surgeries, sacrocolpopexies, gastric bypasses and robot-assisted laparoscopic prostatectomies). Taken together, these findings suggested that the barbed suture significantly shortened the suture time in laparoscopic myomectomies (5.50 min), cosmetic surgeries (6.76 min), sacrocolpopexies (13.60 min), gastric bypasses (11.30 min) and robot-assisted laparoscopic prostatectomies (0.10 min). Thus the effectiveness need be evaluated based on particular surgeries.

In addition, although the overall effect of operative time decreased in barbed groups, a subgroup analysis suggested that only the operative time of laparoscopic myomectomies (2.73 min) and gastric bypasses (11.70 min) were significantly reduced, which was partially consistent with previous studies^3^,^21^ Furthermore, a subgroup analysis also indicated that the use of barbed sutures resulted in less blood loss in laparoscopic myomectomies, which differed from results obtained in a previous study^21^.

Regarding the postoperative complications, the subgroup analysis only indicated that the number of cosmetic surgeries was higher in the barbed suture groups than the control, whereas the pooled results obtained from other surgeries or studies reported no difference. This result may be due to the two studies^14^,^17^ of cosmetic surgeries, both of which had dermal closure performed on one side with the barbed suture and the conventional suture on the opposite side, which increased the risk of surgical site infection. Moreover, previous studies concerning gynecological surgeries reported that bowel obstruction might be attributable to the increased risk of either adhesions or inflammation caused by the barbs entrapped in the novel suture^3^,^21^.

Another concern our meta-analysis focused on is the comparison of different barbed suture types. Compared with the conventional suture, a unidirectional barbed suture decreased the suture and operative times significantly and also demonstrated more postoperative complications, whereas the pooled results of a bidirectional barbed suture did not statistically differ from the control in all outcomes. Thus, the bidirectional barbed suture appeared safer than the unidirectional sutures; although the pooled overall effect indicated no difference. Interestingly, the sensitivity analysis also showed no differences in postoperative complications between the control and either of the barbed groups. The most probable explanation for this result may be that the unidirectional barbed suture required more skillful surgeons. Because such sutures require cuts and re-stitches once suturing errors occurred, this can probably cause more damage to human tissue. Nevertheless, regarding the bidirectional barbed suture, when the barbs in one direction are in the wrong locations, then it can be modified using the other direction to maintain the tension.

Although there are three types of barbed suture commercially available, this study only identified research studies concerning the unidirectional barbed and bidirectional barbed suture; there were no RCTs on humans referring to the third type, Stratafix (STRATAFIX Knotless Tissue Control Devices, Ethicon Inc., Somerville, NJ, USA). Thus, the feasibility and safety among different barbed sutures used in *in vivo* studies should be taken into consideration in the future^22^.

In addition to the favorable outcomes described above from pooled results, numerous other benefits of barbed sutures exist regardless of the patients or surgeons. For example, the barbed suture can eliminate knot tying and the speed of the placement of the sutures. Furthermore, eliminating the need for an assistant’s hand to follow the suture placement, enhancing the equal distribution of tension, and creating the possibility of improved scar cosmoses are also compelling validations for using this state-of-the-art technique.

Our pooled outcome provides convincing evidence for the relationship between the barbed suture and some important surgical indicators. However, caution should be taken to explain the pooled results due to the limitations of our study. (1) Relatively high heterogeneity among studies was estimated for surgical related outcomes, particularly in suture time and estimated blood loss. (2) Although our literature search was extensive, it did not cover conference publications and letters to the editor. (3) There was a lack of cost-effectiveness, cost-benefit, and cost-utility analyses, and the descriptive economic analysis of this study was imperfect. (4) Considering the high heterogeneity of all of the research studies, we performed the SMD for most of the outcomes.

Nevertheless, our results renew a latest meta-analysis on barbed sutures. To the best of our knowledge, this is the most comprehensive meta-analysis to date investigating the association between barbed and traditional sutures.

In conclusion, with the advantages of shorter suture and operative times, postoperative complications were likely to occur more often when using unidirectional barbed sutures. Future studies should also be performed to comprehensively analyze the effect on cost-effectiveness.

## Methods

### Study identification and selection

The MEDLINE, EMBASE and the Cochrane Library databases were searched using the following terms: “barbed” OR “knotless” AND “suturing” OR “suture” (last updated in Feb. 2015). To modify the results and to avoid publication bias, we also searched clinical trials registered in ClinicalTrials.gov (last updated in Feb. 2015).

All studies had to meet the following inclusion criteria: (a) study design had to be a RCT based on human subjects; (b) patients underwent surgical operation; (c) interventions had to be conventional suture vs. barbed suture; and (d) studies should report at least one of the outcomes with detailed data, such as suture time, estimated blood loss, operative time, and postoperative complications. The following exclusion criteria were also applied: (a) conventional sutures were other materials, such as mesh or staple rather than smooth sutures; (b) abstracts or overlapped studies; and (c) studies published in languages other than English. The computer search was supplemented with manual searches for references of included studies.

### Data Extraction and Outcome Measures

We imported the search results into bibliographic citation management software (EndNote X7, Thomson Reuters, USA). Two reviewers independently collected the data and reached a consensus on all items. The following items were extracted from each study if available: first author’s surname, publication year, original country, sample size, type of suture, and postoperative complications.

The main outcome measures chosen for the current meta-analysis were operative time, suture time, estimated blood loss or change in hemoglobin level and postoperative complications. Heterogeneity of the outcomes was assessed to confirm the appropriateness of combining individual studies.

### Definition

Operative time was defined as the total time of surgery. Suture time was defined as the time needed for the completion of the surgical site incision, anastomosis time, and closure time. Estimated blood loss (ml) or change in hemoglobin level (g/dL) (different studies reported different indices of blood loss) was defined as the blood loss during the operation, and it was usually obtained from both the anesthesia records and/or the surgeons’ operative reports. After surgeries, postoperative complications of the suture were also recorded. Both unidirectional and bidirectional barbed sutures were evaluated together as the barbed suture category.

### Methodological Quality Assessment

The risk of bias of included RCTs and was assessed following Cochrane recommendations, considering random sequence generation, allocation concealment, blinding of participants and personnel, blinding of outcome assessment, incomplete outcome data and selective reporting^23^. We searched the protocol of each trial to assess the selective reporting. Publication bias was evaluated using the funnel plot.

### Data Synthesis and Analysis

The studies were divided into seven subgroups according to the seven different surgeries, which were also divided into two subgroups according to the two types of barbed suture; in addition, separate meta-analysis was performed within different subgroups. In all analyses, we estimated the pooled mean difference (MD) and standardized mean difference (SMD) to assess continuous data, while the pooled odds ratios (ORs) were calculated for the assessment of dichotomous data (postoperative complications). The pooled estimations regarding outcomes expressed as either dichotomous or continuous variables were calculated using the random effect model (postoperative complications using fixed effect model). The existence of statistical heterogeneity between the included studies was assessed using the χ^2^ test and I^2^ test. In addition, we also performed sensitivity analyses to examine the robustness of the estimates and assessed the risk of publication bias using Begg’s funnel plots. For all analyses, P < 0.05 was considered statistically significant. Statistical analyses were performed using the software programs Review Manager (Version 5.3).

## Additional Information

**How to cite this article**: Lin, Y. *et al*. The Efficacy and Safety of Knotless Barbed Sutures in the Surgical Field: A Systematic Review and Meta-analysis of Randomized Controlled Trials. *Sci. Rep*. **6**, 23425; doi: 10.1038/srep23425 (2016).

## Supplementary Material

Supplementary Information

## Figures and Tables

**Table 1:** Basic characteristics of all pooled studies in the meta-analysis.

Author/Year	Type of surgery	Country	Barbed type	Sample size (barbed/control)	Cost		Complications
					Barbed	Conventional	
Murtha 2006^5^	Cesarean delivery	USA	B	127/61	NS	NS	Wound dehiscence, incisional infection, surgical complication, seroma, hematoma, others
Alessandri 2010^6^	Laparoscopic myomectomy	Italy	U	22/22	€ 20	€7.30	Ureteric injury, bladder injury, or bowel injury
Naki 2010^7^	Cesarean delivery	Turkey	U	39/39	NS	NS	Wound dehiscence, incisional infection, seroma, hematoma
Williams 2010^8^	Robot-assisted laparoscopic prostatectomy	USA	U	45/36	$51.52	$8.44	cystogram leak
Sammon 2011^9^	Robot-assisted laparoscopic prostatectomy	USA	B	31/33	NS	NS	Leaked urine, urinated blood, had pain or burning with urination
Ting 2012^10^	Arthroplasty	USA	B	31/29	THA:$52.75±$19.96; TKA:$52.84 ±$19.96	THA:$12.79 ±$1.95; TKA:$9.43 ± $1.91,	Wound related or not complications
Zorn 2012^11^	Robot-assisted laparoscopic prostatectomy	Canada	U	33/33	$48.05	$70.25	Urinary retention, clinical urinary VUA leakage, anastomotic stricture, prolonged haematuria (>2 days)
Ardovino 2013^12^	Laparoscopic myomectomy	Italy	B	36/81	NS	NS	Wound dehiscence, bleeding
Ardovino 2013^13^	Laparoscopic hysterectomy	Italy	B	18/43	NS	NS	Bleeding, dyspareunia, and ureteric, bladder, or bowel injury occurred.
Einarsson 2013^14^	Laparoscopic hysterectomy	USA	B	32/31	NS	NS	Dehiscence, infection, bleeding, others
Grigoryants 2013^15^	Comestic surgery	USA	U	30/30	$47 or 94.6	$45.69 or 91.38	Wound infection, wound dehiscence, and suture extrusion,
Milone 2013^16^	Gastric bypass	Italy	U	30/30	€26	€39.9±5.2	Incidence of leak, bleeding, and stenosis
Gililland 2014^17^	Arthroplasty	USA	B	191/203	$324± $118	$419 ±$116	Broken sutures, needle sticks, stitch abscess, cellulitis, lymphangitis, sepsis systemic symptoms, pulmonary embolism
Rubin 2014^18^	Comestic surgery	USA& Germany	U	229/229	NS	NS	Wound dehiscence, suture extrusion, granuloma,and local wound infection
Smith 2014^19^	Arthroplasty	USA	B	18/16	$106.33	$14.4	Superficial wound infections, prominent suture
Tan-Kim 2014^20^	Sacrocolpopexy	USA	B	32/32	$38	$32 – 96	Developed back pain, mesh erosion, vaginal pain
Sah 2015^21^	Arthroplasty	USA	B	50/50	NS	NS	Wound dehiscence or disruption of the arthrotomy, suture irritation, suture end extrusion

B: Bidirectional; U: Unidirectional.

NS: Not stated; THA:total hip arthroplasty;TKA:total knee arthroplasty;USA: the United States of America.

**Table 2:** Pooled outcomes of all the subgroups.

Outcomes	No. of Studies	No. of cases: Barbed/Control	SMD/MD/OR	95%CI	Heterogeneity:	P value for effect size
SUTURE TIME						
Laparoscopic myomectomy¶	2	58/103	−5.50	[−7.03, −3.96]	P = 0.66; I^2^ = 0%	Z = 7.04 (P < 0.00001)
Laparoscopic hysterectomy¶	2	50/74	−1.10	[−4.52, 2.32]	P = 0.02; I^2^ = 83%	Z = 0.63 (P = 0.53)
Arthroplasty¶	3	259/269	−0.66	[−4.43, 3.11]	P < 0.00001; I^2^ = 97%	Z = 0.34 (P = 0.73)
Cosmetic surgery¶	2	259/259	−6.76	[−8.72, −4.79]	P= 0.25; I^2^ = 25%	Z = 6.73 (P < 0.00001)
Sacrocolpopexy¶	1	32/32	−13.60	[−20.63, −6.57]	N/A	Z = 3.79 (P = 0.0001)
Gastric bypass¶	1	30/30	−11.30	[−12.23, −10.37]	N/A	Z =23.73 (P < 0.00001)
Robot-assisted laparoscopic prostatectomy¶	1	45/36	−0.10	[−0.19, −0.01]	N/A	Z = 2.24 (P = 0.03)
Cesarean delivery¶	1	127/61	0.60	[−0.30, 1.50]	N/A	Z = 1.31 (P = 0.19)
Unidirectional barbed§	5	356/347	−1.75	[−2.69, −0.81]	P < 0.00001; I^2^ = 95%	Z = 3.65 (P = 0.0003)
Bidirectional barbed§	7	454/467	−0.28	[−0.89, 0.32]	P < 0.00001; I^2^ = 94%	Z = 0.92 (P = 0.36)
OPERATIVE TIME						
Robot-assisted laparoscopic prostatectomy¶	2	76/69	−6.85	[−14.87, 1.17]	P = 0.90; I^2^ = 0%	Z = 1.68 (P = 0.09)
Laparoscopic myomectomy¶	2	58/103	−2.73	[−5.32, −0.14]	P = 0.43; I^2^ = 0%	Z = 2.07 (P = 0.04)
Laparoscopic hysterectomy¶	2	50/74	−4.48	[−13.40, 4.43]	P = 0.31; I^2^ = 3%	Z = 0.32 (P = 0.99)
Gastric bypass¶	1	30/30	−11.70	[−22.83, −0.57]	N/A	Z = 2.06 (P = 0.04)
Unidirectional barbed§	4	128/121	−0.35	[−0.60, −0.09]	P = 0.85; I^2^ = 0%	Z = 2.70 (P = 0.007)
Bidirectional barbed§	3	86/155	−0.20	[−0.55, 0.16]	P = 0.19; I^2^ = 39%	Z = 1.09 (P = 0.28)
ESTIMATE THE INTRAOPERATIVE BLOOD LOSS						
Robot-assisted laparoscopic prostatectomy§	2	78/69	0.03	[−0.29, 0.36]	P = 0.55; I^2^ = 0%	Z = 0.19 (P = 0.85)
Laparoscopic myomectomy§	1	22/22	−0.83	[−1.45, −0.21]	N/A	Z = 2.64 (P = 0.008)
Laparoscopic hysterectomy§	1	32/31	0.31	[−0.18, 0.81]	N/A	Z = 1.23 (P = 0.22)
Unidirectional barbed§	3	100/91	−0.22	[−0.74, 0.29]	P = 0.04; I^2^ = 68%	Z = 0.85 (P = 0.40)
Bidirectional barbed§	1	32/31	0.31	[−0.18, 0.81]	N/A	Z = 1.23 (P = 0.22)
COMPLICATIONS						
Robot-assisted laparoscopic prostatectomy*	3	109/102	2.79	[0.89, 8.79]	P = 0.10; I^2^ = 62%	Z = 1.75 (P = 0.08)
Laparoscopic myomectomy*	2	58/103	N/A	N/A	N/A	N/A
Laparoscopic hysterectomy*	2	50/74	0.70	[0.24, 2.08]	N/A	Z = 0.63 (P = 0.53)
Cesarean delivery*	2	166/100	0.69	[0.34, 1.38]	P = 0.26; I^2^ = 20%	Z = 1.05 (P = 0.29)
Arthroplasty*	4	290/298	1.19	[0.58, 2.41]	P = 0.12; I^2^ = 48%	Z = 0.48 (P = 0.63)
Cosmetic surgery*	2	259/259	2.47	[1.50, 4.06]	P = 0.01; I^2^ = 83%	Z = 3.56 (P = 0.0004)
Gastric bypass*	1	30/30	0.50	[0.05, 5.02]	N/A	Z = 0.59 (P = 0.56)
Sacrocolpopexy*	1	32/32	1.53	[0.25, 9.38]	N/A	Z = 0.46 (P = 0.64)
Unidirectional barbed*	7	428/419	2.13	[1.35, 3.35]	P = 0.007; I^2^ = 72%	Z = 3.25 (P = 0.001)
Bidirectional barbed*	9	516/529	0.96	[0.61, 1.50]	P=0.63; I^2^ = 0%	Z = 0.17 (P = 0.86)

^¶^MD= mean difference.

^§^SMD=standardized mean difference.

^*^OR=Odds ratio.

NA: Not applicable.
