# Supplementary material for: The Efficacy and Safety of Knotless Barbed Sutures in the Surgical Field: A Systematic Review and Meta-analysis of Randomized Controlled Trials
Source: Sci Rep. 2016 Mar 23;6:23425. doi: 10.1038/srep23425 (PMC4804241; doi:10.1038/srep23425)
Supplement: Supplementary Information [file srep23425-s1.pdf]

Title: The Efficacy and Safety of Knotless Barbed Sutures in the Surgical Field: A Systematic Review and Meta-analysis of Randomized Controlled Trials

Authors

Yifei Lin [1], Sike Lai [2], Liang Du [3*], Jin Huang [3*]

1. Institute of Urology, Department of Urology, West China Hospital, Sichuan University, Guoxuexiang 37, Chengdu, Sichuan, 610041, China
2. West China School of Medicine, Sichuan University, Guoxuexiang 37, Chengdu, Sichuan, 610041, China.
3. West China Hospital, Sichuan University, Guoxuexiang 37, Chengdu, Sichuan, 610041, China.

* Correspondence author

Correspondence should be addressed to
1. Dr. Jin HUANG at West China Hospital, Sichuan University, Guoxuexiang 37 Chengdu, Sichuan 610041, China with E-mail address: michael_huangjin@163.com
2. Dr. Liang DU at West China Hospital, Sichuan University, Guoxuexiang 37, Chengdu, Sichuan, China with E-mail address: 125798620@qq.com

Supplementary Figure Legends:

Appendix Figure 1: A forest plot of suturing time of different surgeries with or without barbed suture

Appendix Figure 2: A forest plot of suturing time of different barbed types with or without barbed suture

Appendix Figure 3: A forest plot of operative time of different surgeries with or without barbed suture

Appendix Figure 4: A forest plot of operative time of different barbed types with or without barbed suture

Appendix Figure 5: A forest plot of estimated blood loss of different surgeries with or without barbed suture

Appendix Figure 6: A forest plot of estimated blood loss of different barbed types with or without barbed suture

Appendix Figure 7: A forest plot of postoperative complications of different surgeries with or without barbed suture

Appendix Figure 8: A forest plot of postoperative complications of different barbed types with or without barbed suture

Appendix Figure 9: Sensitivity analyses of postoperative complications of different barbed types with or without barbed suture

Appendix Figure 10: Funnel plot of suturing time in all included studies

Appendix Figure 11: Funnel plot of operative time in all included studies

Appendix Figure 12: Funnel plot of estimate blood loss in all included studies

Appendix Figure 13: Funnel plot of postoperative complications in all included studies

Appendix Table 1: Quality assessment of studies in the meta-analysis based on Cochrane recommendations

Appendix Figure 1: A forest plot of suturing time of different surgeries with or without barbed suture

Study or Subgroup	Barbed Mean	Barbed SD	Barbed Total	Control Mean	Control SD	Control Total	Weight	Mean Difference IV, Random, 95% CI	Year
**1.1.1 Laparoscopic myomectomy**									
Alessandri 2010	11.5	4.1	22	17.4	3.8	22	7.8%	-5.90 [-8.24, -3.56]	2010
Ardovino 2013A	6.6	4.7	36	11.8	6.1	81	7.9%	-5.20 [-7.23, -3.17]	2013
**Subtotal (95% CI)**			**58**			**103**	**15.7%**	**-5.50 [-7.03, -3.97]**	
Heterogeneity: Tau² = 0.00; Chi² = 0.20, df = 1 (P = 0.66); I² = 0%									
Test for overall effect: Z = 7.04 (P < 0.00001)									
**1.1.2 Laparoscopic hysterectomy**									
Ardovino 2013B	3.9	2	18	6.6	3.7	43	8.1%	-2.70 [-4.14, -1.26]	2013
Einarsson 2013	10.4	5.2	32	9.6	4.8	31	7.7%	0.80 [-1.67, 3.27]	2013
**Subtotal (95% CI)**			**50**			**74**	**15.9%**	**-1.10 [-4.52, 2.32]**	
Heterogeneity: Tau² = 5.06; Chi² = 5.75, df = 1 (P = 0.02); I² = 83%									
Test for overall effect: Z = 0.63 (P = 0.53)									
**1.1.3 Arthroplasty**									
Gililland 2014	9.8	4.22	191	14.4	3.98	203	8.3%	-4.60 [-5.41, -3.79]	2014
Smith 2014	26.5	6.83	18	16.78	3.28	16	7.1%	9.72 [6.18, 13.26]	2014
Sah 2015	11.4	2.2	50	16.1	2.2	50	8.3%	-4.70 [-5.56, -3.84]	2015
**Subtotal (95% CI)**			**259**			**269**	**23.7%**	**-0.66 [-4.43, 3.11]**	
Heterogeneity: Tau² = 10.04; Chi² = 61.55, df = 2 (P < 0.00001); I² = 97%									
Test for overall effect: Z = 0.34 (P = 0.73)									
**1.1.4 Cosmetic surgery**									
Grigoryants 2013	7.87	12.4	30	12.36	1.72	30	6.5%	-4.49 [-8.97, -0.01]	2013
Rubin 2014	12	4.6	229	19.2	6.7	229	8.3%	-7.20 [-8.25, -6.15]	2014
**Subtotal (95% CI)**			**259**			**259**	**14.8%**	**-6.76 [-8.72, -4.79]**	
Heterogeneity: Tau² = 0.92; Chi² = 1.33, df = 1 (P = 0.25); I² = 25%									
Test for overall effect: Z = 6.73 (P < 0.00001)									
**1.1.5 Sacrocolpopexy**									
Tan-Kim 2014	28.7	12.6	32	42.3	15.9	32	5.0%	-13.60 [-20.63, -6.57]	2014
**Subtotal (95% CI)**			**32**			**32**	**5.0%**	**-13.60 [-20.63, -6.57]**	
Heterogeneity: Not applicable									
Test for overall effect: Z = 3.79 (P = 0.0001)									
**1.1.6 Gastric bypass**									
Milone 2013	12.8	1.4	30	24.1	2.2	30	8.3%	-11.30 [-12.23, -10.37]	2013
**Subtotal (95% CI)**			**30**			**30**	**8.3%**	**-11.30 [-12.23, -10.37]**	
Heterogeneity: Not applicable									
Test for overall effect: Z = 23.73 (P < 0.00001)									
**1.1.7 Robot-assisted laparoscopic prostatectomy**									
Williams 2010	9.7	0.2	45	9.8	0.2	36	8.4%	-0.10 [-0.19, -0.01]	2010
**Subtotal (95% CI)**			**45**			**36**	**8.4%**	**-0.10 [-0.19, -0.01]**	
Heterogeneity: Not applicable									
Test for overall effect: Z = 2.24 (P = 0.03)									
**1.1.8 Cesarean delivery**									
Murtha 2006	9.5	3.22	127	8.9	2.81	61	8.3%	0.60 [-0.30, 1.50]	2006
**Subtotal (95% CI)**			**127**			**61**	**8.3%**	**0.60 [-0.30, 1.50]**	
Heterogeneity: Not applicable									
Test for overall effect: Z = 1.31 (P = 0.19)									
**Total (95% CI)**			**860**			**864**	**100.0%**	**-3.56 [-6.01, -1.12]**	
Heterogeneity: Tau² = 18.61; Chi² = 1033.05, df = 12 (P < 0.00001); I² = 99%									
Test for overall effect: Z = 2.85 (P = 0.004)									
Test for subgroup differences: Chi² = 654.28, df = 7 (P < 0.00001), I² = 98.9%									

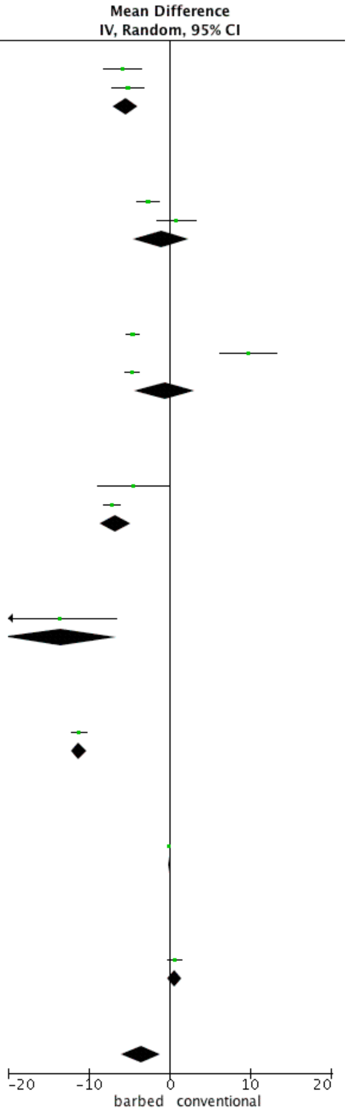

Appendix Figure 2: A forest plot of suturing time of different barbed types with or without barbed suture

Study or Subgroup	Barbed Mean	Barbed SD	Barbed Total	Control Mean	Control SD	Control Total	Weight	Std. Mean Difference IV, Random, 95% CI	Year
**1.2.1 Unidirectional barbed**									
Williams 2010	9.7	0.2	45	9.8	0.2	36	8.0%	-0.50 [-0.94, -0.05]	2010
Alessandri 2010	11.5	4.1	22	17.4	3.8	22	7.4%	-1.47 [-2.14, -0.79]	2010
Milone 2013	12.8	1.4	30	24.1	2.2	30	5.5%	-6.05 [-7.28, -4.82]	2013
Grigoryants 2013	7.87	12.4	30	12.36	1.72	30	7.8%	-0.50 [-1.02, 0.01]	2013
Rubin 2014	12	4.6	229	19.2	6.7	229	8.4%	-1.25 [-1.45, -1.05]	2014
**Subtotal (95% CI)**			**356**			**347**	**37.1%**	**-1.75 [-2.69, -0.81]**	
Heterogeneity: Tau² = 1.04; Chi² = 77.26, df = 4 (P < 0.00001); I² = 95%									
Test for overall effect: Z = 3.65 (P = 0.0003)									
**1.2.2 Bidirectional barbed**									
Murtha 2006	9.5	3.22	127	8.9	2.81	61	8.3%	0.19 [-0.11, 0.50]	2006
Einarsson 2013	10.4	5.2	32	9.6	4.8	31	7.9%	0.16 [-0.34, 0.65]	2013
Ardovino 2013B	3.9	2	18	6.6	3.7	43	7.7%	-0.81 [-1.38, -0.24]	2013
Ardovino 2013A	6.6	4.7	36	11.8	6.1	81	8.1%	-0.90 [-1.31, -0.49]	2013
Gililland 2014	9.8	4.22	191	14.4	3.98	203	8.4%	-1.12 [-1.33, -0.91]	2014
Tan-Kim 2014	28.7	12.6	32	42.3	15.9	32	7.8%	-0.94 [-1.45, -0.42]	2014
Smith 2014	26.5	6.83	18	16.78	3.28	16	6.9%	1.74 [0.93, 2.54]	2014
Sah 2015	11.4	2.2	50	16.1	2.2	50	7.9%	-2.12 [-2.61, -1.63]	2015
**Subtotal (95% CI)**			**504**			**517**	**62.9%**	**-0.51 [-1.13, 0.11]**	
Heterogeneity: Tau² = 0.74; Chi² = 128.27, df = 7 (P < 0.00001); I² = 95%									
Test for overall effect: Z = 1.60 (P = 0.11)									
**Total (95% CI)**			**860**			**864**	**100.0%**	**-0.95 [-1.43, -0.46]**	
Heterogeneity: Tau² = 0.71; Chi² = 223.09, df = 12 (P < 0.00001); I² = 95%									
Test for overall effect: Z = 3.83 (P = 0.0001)									
Test for subgroup differences: Chi² = 4.67, df = 1 (P = 0.03), I² = 78.6%									

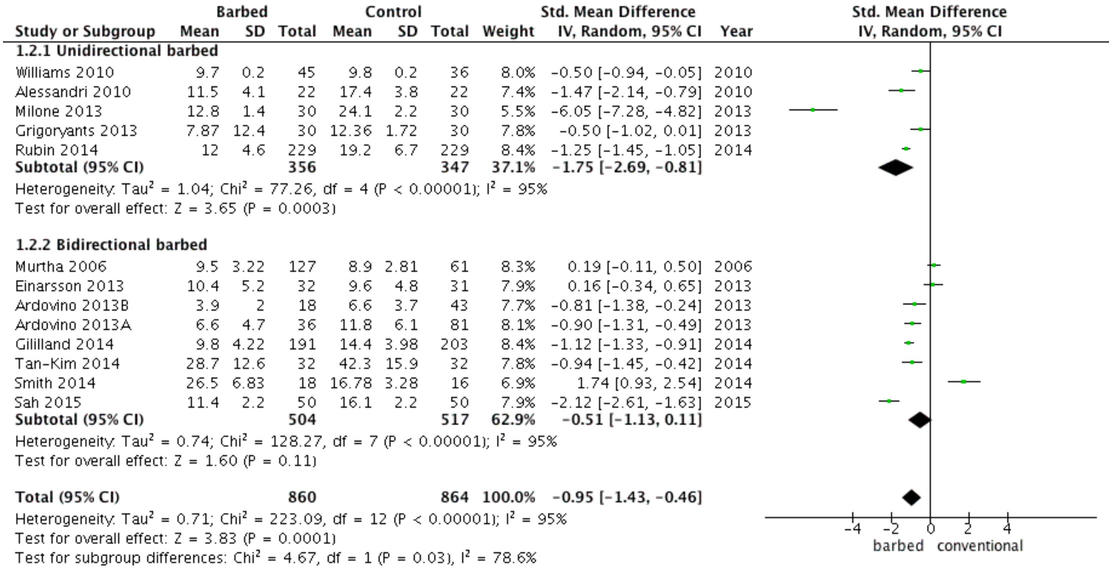

Appendix Figure 3: A forest plot of operative time of different surgeries with or without barbed suture

Study or Subgroup	barbed Mean	barbed SD	barbed Total	Control Mean	Control SD	Control Total	Weight	Mean Difference IV, Random, 95% CI
**2.1.1 Robot-assisted laparoscopic prostatectomy**								
Williams 2010	103.8	21.2	45	110.4	19.4	36	6.8%	-6.60 [-15.46, 2.26]
Zorn 2012	166	41	33	174	37	33	1.5%	-8.00 [-26.84, 10.84]
**Subtotal (95% CI)**			**78**			**69**	**8.2%**	**-6.85 [-14.87, 1.17]**
Heterogeneity: Tau² = 0.00; Chi² = 0.02, df = 1 (P = 0.90); I² = 0%								
Test for overall effect: Z = 1.68 (P = 0.09)								
**2.1.2 Laparoscopic myomectomy**								
Alessandri 2010	73.3	21.4	22	80.7	18.6	22	3.8%	-7.40 [-19.25, 4.45]
Ardovino 2013A	36.8	6.7	36	39.3	6.9	81	75.2%	-2.50 [-5.15, 0.15]
**Subtotal (95% CI)**			**58**			**103**	**79.0%**	**-2.73 [-5.32, -0.14]**
Heterogeneity: Tau² = 0.00; Chi² = 0.63, df = 1 (P = 0.43); I² = 0%								
Test for overall effect: Z = 2.07 (P = 0.04)								
**2.1.3 Laparoscopic hysterectomy**								
Ardovino 2013B	131.5	14.8	18	137.2	14.7	43	8.0%	-5.70 [-13.83, 2.43]
Einarsson 2013	146.4	76.5	32	133.9	61.3	31	0.5%	12.50 [-21.68, 46.68]
**Subtotal (95% CI)**			**50**			**74**	**8.5%**	**-4.48 [-13.40, 4.43]**
Heterogeneity: Tau² = 4.97; Chi² = 1.03, df = 1 (P = 0.31); I² = 3%								
Test for overall effect: Z = 0.99 (P = 0.32)								
**2.1.4 Gastric bypass**								
Milone 2013	122.7	31.1	30	134.4	0.8	30	4.3%	-11.70 [-22.83, -0.57]
**Subtotal (95% CI)**			**30**			**30**	**4.3%**	**-11.70 [-22.83, -0.57]**
Heterogeneity: Not applicable								
Test for overall effect: Z = 2.06 (P = 0.04)								
**Total (95% CI)**			**216**			**276**	**100.0%**	**-3.63 [-5.93, -1.32]**
Heterogeneity: Tau² = 0.00; Chi² = 4.85, df = 6 (P = 0.56); I² = 0%								
Test for overall effect: Z = 3.09 (P = 0.002)								
Test for subgroup differences: Chi² = 3.13, df = 3 (P = 0.37), I² = 4.2%								

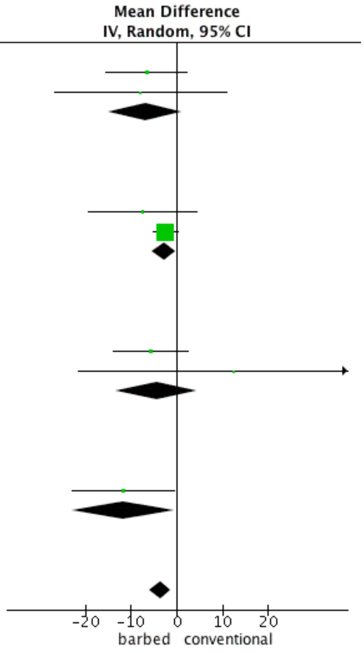

Appendix Figure 4: A forest plot of operative time of different barbed types with or without barbed suture

Study or Subgroup	barbed Mean	barbed SD	barbed Total	Control Mean	Control SD	Control Total	Weight	Std. Mean Difference IV, Random, 95% CI
**2.2.1 Unidirectional barbed**								
Alessandri 2010	73.3	21.4	22	80.7	18.6	22	9.5%	-0.36 [-0.96, 0.23]
Milone 2013	122.7	31.1	30	134.4	0.8	30	12.7%	-0.52 [-1.04, -0.01]
Williams 2010	103.8	21.2	45	110.4	19.4	36	17.3%	-0.32 [-0.76, 0.12]
Zorn 2012	166	41	33	174	37	33	14.4%	-0.20 [-0.69, 0.28]
**Subtotal (95% CI)**			**130**			**121**	**53.8%**	**-0.34 [-0.59, -0.09]**
Heterogeneity: Tau² = 0.00; Chi² = 0.82, df = 3 (P = 0.85); I² = 0%								
Test for overall effect: Z = 2.70 (P = 0.007)								
**2.2.2 Bidirectional barbed**								
Ardovino 2013A	36.8	6.7	36	39.3	6.9	81	21.5%	-0.36 [-0.76, 0.03]
Ardovino 2013B	131.5	14.8	18	137.2	14.7	43	10.9%	-0.38 [-0.94, 0.17]
Einarsson 2013	146.4	76.5	32	133.9	61.3	31	13.7%	0.18 [-0.32, 0.67]
**Subtotal (95% CI)**			**86**			**155**	**46.2%**	**-0.20 [-0.55, 0.16]**
Heterogeneity: Tau² = 0.04; Chi² = 3.30, df = 2 (P = 0.19); I² = 39%								
Test for overall effect: Z = 1.09 (P = 0.28)								
**Total (95% CI)**			**216**			**276**	**100.0%**	**-0.28 [-0.46, -0.10]**
Heterogeneity: Tau² = 0.00; Chi² = 4.66, df = 6 (P = 0.59); I² = 0%								
Test for overall effect: Z = 3.00 (P = 0.003)								
Test for subgroup differences: Chi² = 0.45, df = 1 (P = 0.50), I² = 0%								

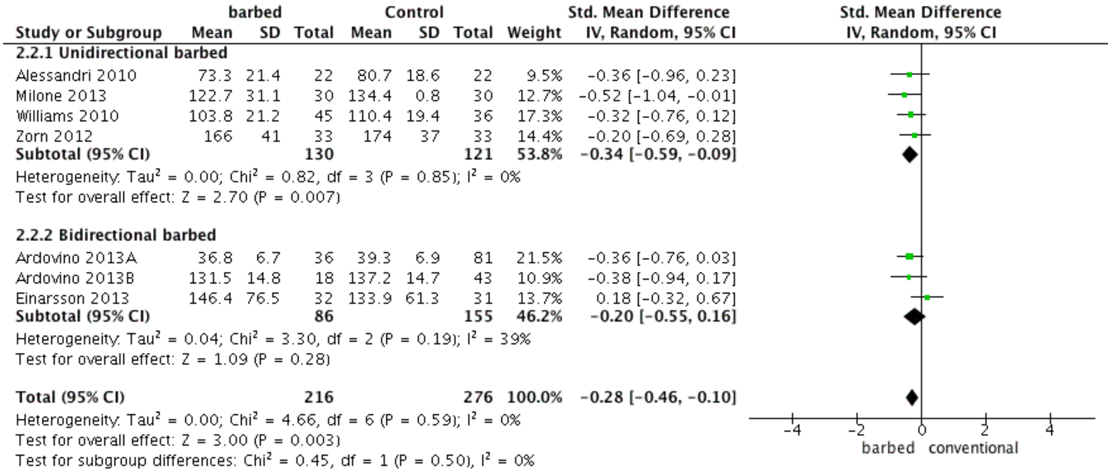

Appendix Figure 5: A forest plot of estimated blood loss of different surgeries with or without barbed suture

Study or Subgroup	Barbed Mean	Barbed SD	Barbed Total	Control Mean	Control SD	Control Total	Weight	Std. Mean Difference IV, Random, 95% CI
**3.1.1 Robot-assisted laparoscopic prostatectomy**								
Williams 2010	181.5	78.1	45	173.3	49.7	36	27.4%	0.12 [-0.32, 0.56]
Zorn 2012	167	95	33	175	110	33	25.8%	-0.08 [-0.56, 0.41]
**Subtotal (95% CI)**			**78**			**69**	**53.2%**	**0.03 [-0.29, 0.36]**
Heterogeneity: Tau² = 0.00; Chi² = 0.35, df = 1 (P = 0.55); I² = 0%								
Test for overall effect: Z = 0.19 (P = 0.85)								
**3.1.2 Laparoscopic myomectomy**								
Alessandri 2010	0.6	0.3	22	0.9	0.4	22	21.4%	-0.83 [-1.45, -0.21]
**Subtotal (95% CI)**			**22**			**22**	**21.4%**	**-0.83 [-1.45, -0.21]**
Heterogeneity: Not applicable								
Test for overall effect: Z = 2.64 (P = 0.008)								
**3.1.3 Laparoscopic hysterectomy**								
Einarsson 2013	119.5	112.5	32	89.7	69.9	31	25.4%	0.31 [-0.18, 0.81]
**Subtotal (95% CI)**			**32**			**31**	**25.4%**	**0.31 [-0.18, 0.81]**
Heterogeneity: Not applicable								
Test for overall effect: Z = 1.23 (P = 0.22)								
**Total (95% CI)**			**132**			**122**	**100.0%**	**-0.09 [-0.52, 0.35]**
Heterogeneity: Tau² = 0.13; Chi² = 8.80, df = 3 (P = 0.03); I² = 66%								
Test for overall effect: Z = 0.39 (P = 0.70)								
Test for subgroup differences: Chi² = 8.44, df = 2 (P = 0.01), I² = 76.3%								

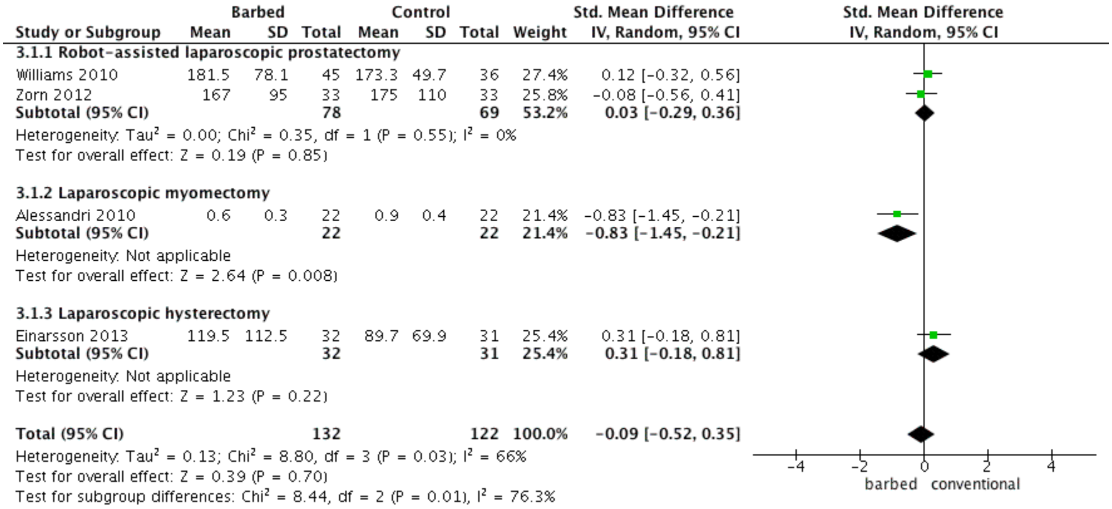

Appendix Figure 6: A forest plot of estimated blood loss of different barbed types with or without barbed suture

Study or Subgroup	Barbed Mean	Barbed SD	Barbed Total	Control Mean	Control SD	Control Total	Weight	Std. Mean Difference IV, Random, 95% CI
**3.2.1 Unidirectional barbed**								
Alessandri 2010	0.6	0.3	22	0.9	0.4	22	21.4%	-0.83 [-1.45, -0.21]
Williams 2010	181.5	78.1	45	173.3	49.7	36	27.4%	0.12 [-0.32, 0.56]
Zorn 2012	167	95	33	175	110	33	25.8%	-0.08 [-0.56, 0.41]
**Subtotal (95% CI)**			**100**			**91**	**74.6%**	**-0.22 [-0.74, 0.29]**
Heterogeneity: Tau² = 0.14; Chi² = 6.24, df = 2 (P = 0.04); I² = 68%								
Test for overall effect: Z = 0.85 (P = 0.40)								
**3.2.2 Bidirectional barbed**								
Einarsson 2013	119.5	112.5	32	89.7	69.9	31	25.4%	0.31 [-0.18, 0.81]
**Subtotal (95% CI)**			**32**			**31**	**25.4%**	**0.31 [-0.18, 0.81]**
Heterogeneity: Not applicable								
Test for overall effect: Z = 1.23 (P = 0.22)								
**Total (95% CI)**			**132**			**122**	**100.0%**	**-0.09 [-0.52, 0.35]**
Heterogeneity: Tau² = 0.13; Chi² = 8.80, df = 3 (P = 0.03); I² = 66%								
Test for overall effect: Z = 0.39 (P = 0.70)								
Test for subgroup differences: Chi² = 2.15, df = 1 (P = 0.14), I² = 53.4%								

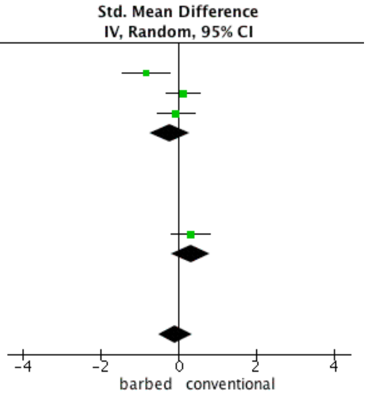

Appendix Figure 7: A forest plot of postoperative complications of different surgeries with or without barbed suture

Study or Subgroup	barbed Events	barbed Total	Control Events	Control Total	Weight	Peto Odds Ratio Peto, Fixed, 95% CI
**4.1.1 Robot-assisted laparoscopic prostatectomy**						
Sammon 2011	1	31	2	33	1.9%	0.54 [0.05, 5.35]
Williams 2010	9	45	1	36	5.6%	4.82 [1.28, 18.10]
Zorn 2012	0	33	0	33		Not estimable
**Subtotal (95% CI)**		**109**		**102**	**7.4%**	**2.79 [0.89, 8.79]**
Total events	10		3			
Heterogeneity: $Chi^2 = 2.63$, df = 1 (P = 0.10); $I^2 = 62\%$						
Test for overall effect: Z = 1.75 (P = 0.08)						
**4.1.2 Laparoscopic myomectomy**						
Alessandri 2010	0	22	0	22		Not estimable
Ardovino 2013A	0	36	0	81		Not estimable
**Subtotal (95% CI)**		**58**		**103**		**Not estimable**
Total events	0		0			
Heterogeneity: Not applicable						
Test for overall effect: Not applicable						
**4.1.3 Laparoscopic hysterectomy**						
Ardovino 2013B	0	18	0	43		Not estimable
Einarsson 2013	8	32	10	31	8.3%	0.70 [0.24, 2.08]
**Subtotal (95% CI)**		**50**		**74**	**8.3%**	**0.70 [0.24, 2.08]**
Total events	8		10			
Heterogeneity: Not applicable						
Test for overall effect: Z = 0.63 (P = 0.53)						
**4.1.4 Cesarean delivery**						
Murtha 2006	22	127	12	61	15.7%	0.85 [0.39, 1.88]
Naki 2010	2	39	6	39	4.6%	0.33 [0.08, 1.42]
**Subtotal (95% CI)**		**166**		**100**	**20.3%**	**0.69 [0.34, 1.38]**
Total events	24		18			
Heterogeneity: $Chi^2 = 1.25$, df = 1 (P = 0.26); $I^2 = 20\%$						
Test for overall effect: Z = 1.05 (P = 0.29)						
**4.1.5 Arthroplasty**						
Gililland 2014	7	191	5	203	7.4%	1.50 [0.48, 4.73]
Sah 2015	0	50	4	50	2.5%	0.13 [0.02, 0.93]
Smith 2014	3	18	1	16	2.3%	2.65 [0.34, 20.76]
Ting 2012	9	31	6	29	7.3%	1.55 [0.49, 4.94]
**Subtotal (95% CI)**		**290**		**298**	**19.5%**	**1.19 [0.58, 2.41]**
Total events	19		16			
Heterogeneity: $Chi^2 = 5.79$, df = 3 (P = 0.12); $I^2 = 48\%$						
Test for overall effect: Z = 0.48 (P = 0.63)						
**4.1.6 Cosmetic surgery**						
Grigoryants 2013	1	30	4	30	3.0%	0.28 [0.04, 1.70]
Rubin 2014	49	229	18	229	36.6%	2.95 [1.76, 4.95]
**Subtotal (95% CI)**		**259**		**259**	**39.6%**	**2.47 [1.50, 4.06]**
Total events	50		22			
Heterogeneity: $Chi^2 = 6.05$, df = 1 (P = 0.01); $I^2 = 83\%$						
Test for overall effect: Z = 3.56 (P = 0.0004)						
**4.1.7 Gastric bypass**						
Milone 2013	1	30	2	30	1.8%	0.50 [0.05, 5.02]
**Subtotal (95% CI)**		**30**		**30**	**1.8%**	**0.50 [0.05, 5.02]**
Total events	1		2			
Heterogeneity: Not applicable						
Test for overall effect: Z = 0.59 (P = 0.56)						
**4.1.8 Sacrocolpopexy**						
Tan-Kim 2014	3	32	2	32	3.0%	1.53 [0.25, 9.38]
**Subtotal (95% CI)**		**32**		**32**	**3.0%**	**1.53 [0.25, 9.38]**
Total events	3		2			
Heterogeneity: Not applicable						
Test for overall effect: Z = 0.46 (P = 0.64)						
**Total (95% CI)**		**994**		**998**	**100.0%**	**1.44 [1.05, 1.97]**
Total events	115		73			
Heterogeneity: $Chi^2 = 28.57$, df = 12 (P = 0.005); $I^2 = 58\%$						
Test for overall effect: Z = 2.27 (P = 0.02)						
Test for subgroup differences: $Chi^2 = 12.86$, df = 6 (P = 0.05), $I^2 = 53.4\%$						

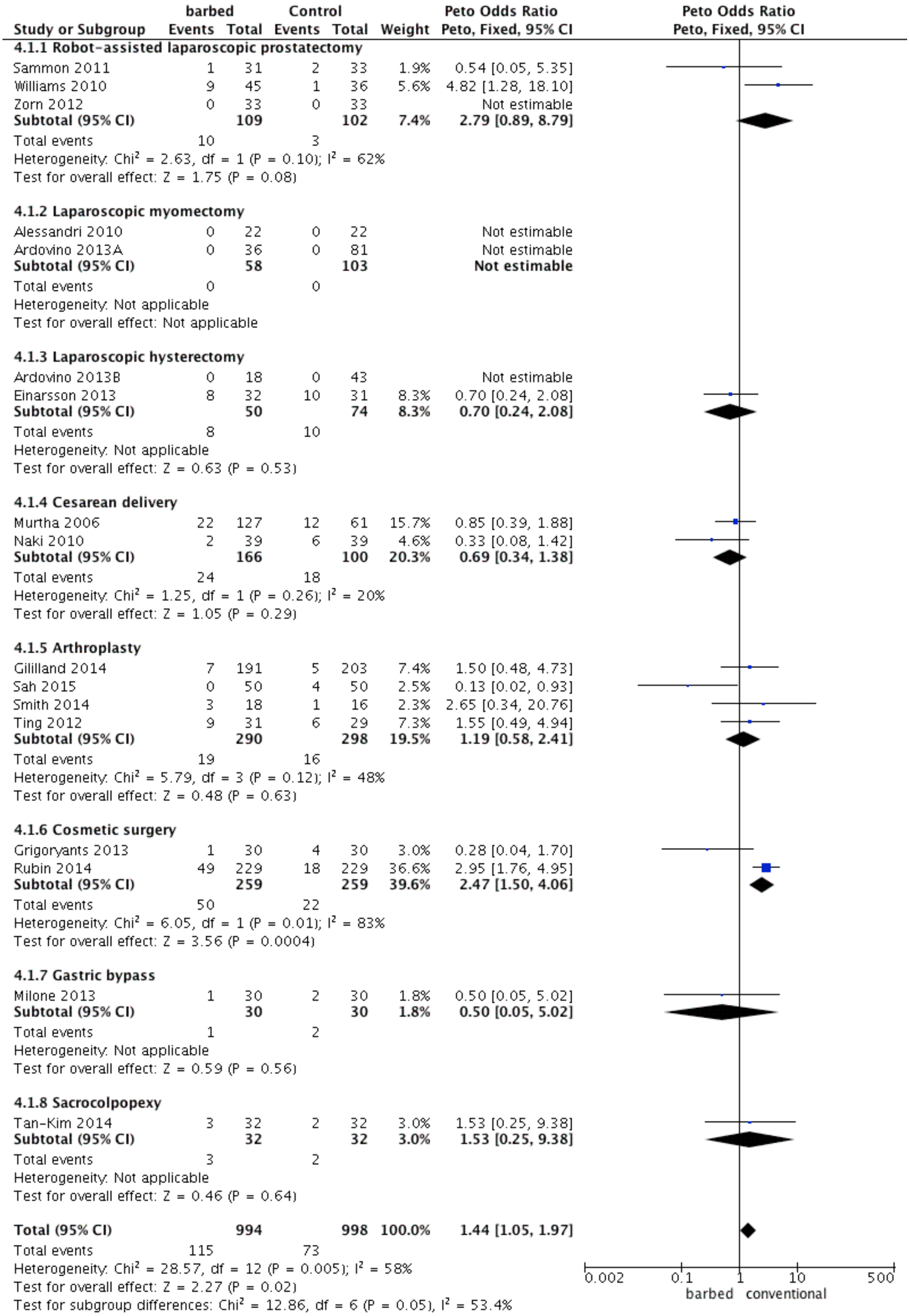

Appendix Figure 8: A forest plot of postoperative complications of different barbed types with or without barbed suture

	barbed		Control			Odds Ratio
Study or Subgroup	Events	Total	Events	Total	Weight	M-H, Fixed, 95% CI
**4.2.1 unidirectional barbed**						
Alessandri 2010	0	22	0	22		Not estimable
Grigoryants 2013	1	30	4	30	5.9%	0.22 [0.02, 2.14]
Milone 2013	1	30	2	30	2.9%	0.48 [0.04, 5.63]
Naki 2010	2	39	6	39	8.7%	0.30 [0.06, 1.58]
Rubin 2014	49	229	18	229	21.6%	3.19 [1.79, 5.67]
Williams 2010	9	45	1	36	1.4%	8.75 [1.05, 72.73]
Zorn 2012	0	33	0	33		Not estimable
**Subtotal (95% CI)**		**428**		**419**	**40.4%**	**2.13 [1.35, 3.35]**
Total events	62		31			
Heterogeneity: Chi² = 14.20, df = 4 (P = 0.007); I² = 72%						
Test for overall effect: Z = 3.25 (P = 0.001)						
**4.2.2 bidirectional barbed**						
Ardovino 2013A	0	36	0	81		Not estimable
Ardovino 2013B	0	18	0	43		Not estimable
Einarsson 2013	8	32	10	31	11.6%	0.70 [0.23, 2.10]
Gililland 2014	7	191	5	203	7.1%	1.51 [0.47, 4.83]
Murtha 2006	22	127	12	61	20.4%	0.86 [0.39, 1.87]
Sah 2015	0	50	4	50	6.8%	0.10 [0.01, 1.95]
Sammon 2011	1	31	2	33	2.9%	0.52 [0.04, 6.00]
Smith 2014	3	18	1	16	1.3%	3.00 [0.28, 32.21]
Tan-Kim 2014	3	32	2	32	2.8%	1.55 [0.24, 9.97]
Ting 2012	9	31	6	29	6.7%	1.57 [0.48, 5.14]
**Subtotal (95% CI)**		**566**		**579**	**59.6%**	**0.96 [0.61, 1.50]**
Total events	53		42			
Heterogeneity: Chi² = 5.23, df = 7 (P = 0.63); I² = 0%						
Test for overall effect: Z = 0.17 (P = 0.86)						
**Total (95% CI)**		**994**		**998**	**100.0%**	**1.43 [1.05, 1.96]**
Total events	115		73			
Heterogeneity: Chi² = 24.47, df = 12 (P = 0.02); I² = 51%						
Test for overall effect: Z = 2.24 (P = 0.03)						
Test for subgroup differences: Chi² = 5.94, df = 1 (P = 0.01), I² = 83.2%						

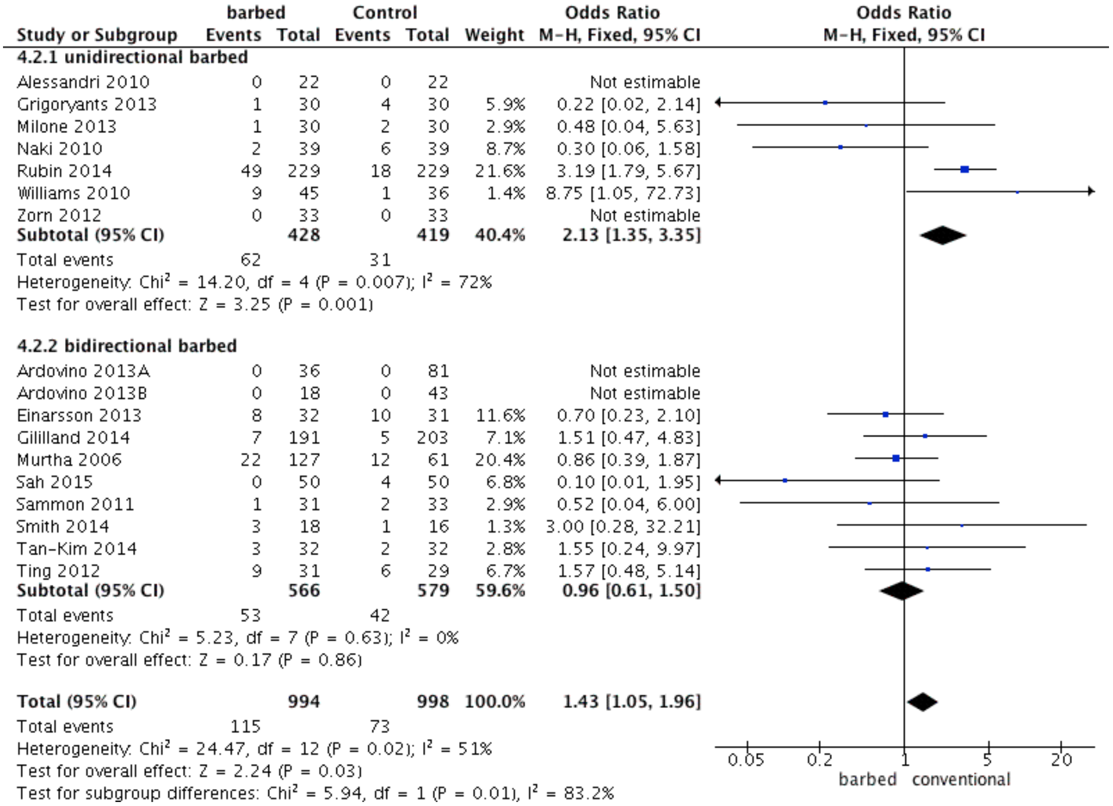

Appendix Figure 9: Sensitivity analyses of postoperative complications of different barbed types with or without barbed suture

Study or Subgroup	barbed Events	barbed Total	Control Events	Control Total	Weight	Odds Ratio M-H, Fixed, 95% CI
**4.2.1 unidirectional barbed**						
Alessandri 2010	0	22	0	22		Not estimable
Grigoryants 2013	1	30	4	30	7.6%	0.22 [0.02, 2.14]
Milone 2013	1	30	2	30	3.8%	0.48 [0.04, 5.63]
Naki 2010	2	39	6	39	11.2%	0.30 [0.06, 1.58]
Rubin 2014	49	229	18	229	0.0%	2.95 [1.76, 4.95]
Williams 2010	9	45	1	36	0.0%	4.82 [1.28, 18.10]
Zorn 2012	0	33	0	33		Not estimable
**Subtotal (95% CI)**		**154**		**154**	**22.7%**	**0.30 [0.09, 0.98]**
Total events	4		12			
Heterogeneity: Chi² = 0.21, df = 2 (P = 0.90); I² = 0%						
Test for overall effect: Z = 2.00 (P = 0.05)						
**4.2.2 bidirectional barbed**						
Ardovino 2013A	0	36	0	81		Not estimable
Ardovino 2013B	0	18	0	43		Not estimable
Einarsson 2013	8	32	10	31	15.1%	0.70 [0.23, 2.10]
Gililand 2014	7	191	5	203	9.2%	1.51 [0.47, 4.83]
Murtha 2006	22	127	12	61	26.5%	0.86 [0.39, 1.87]
Sah 2015	0	50	4	50	8.8%	0.10 [0.01, 1.95]
Sammon 2011	1	31	2	33	3.7%	0.52 [0.04, 6.00]
Smith 2014	3	18	1	16	1.7%	3.00 [0.28, 32.21]
Tan-Kim 2014	3	32	2	32	3.6%	1.55 [0.24, 9.97]
Ting 2012	9	31	6	29	8.7%	1.57 [0.48, 5.14]
**Subtotal (95% CI)**		**566**		**579**	**77.3%**	**0.96 [0.61, 1.50]**
Total events	53		42			
Heterogeneity: Chi² = 5.23, df = 7 (P = 0.63); I² = 0%						
Test for overall effect: Z = 0.17 (P = 0.86)						
**Total (95% CI)**		**720**		**733**	**100.0%**	**0.81 [0.54, 1.22]**
Total events	57		54			
Heterogeneity: Chi² = 8.82, df = 10 (P = 0.55); I² = 0%						
Test for overall effect: Z = 0.99 (P = 0.32)						
Test for subgroup differences: Chi² = 3.25, df = 1 (P = 0.07), I² = 69.3%						

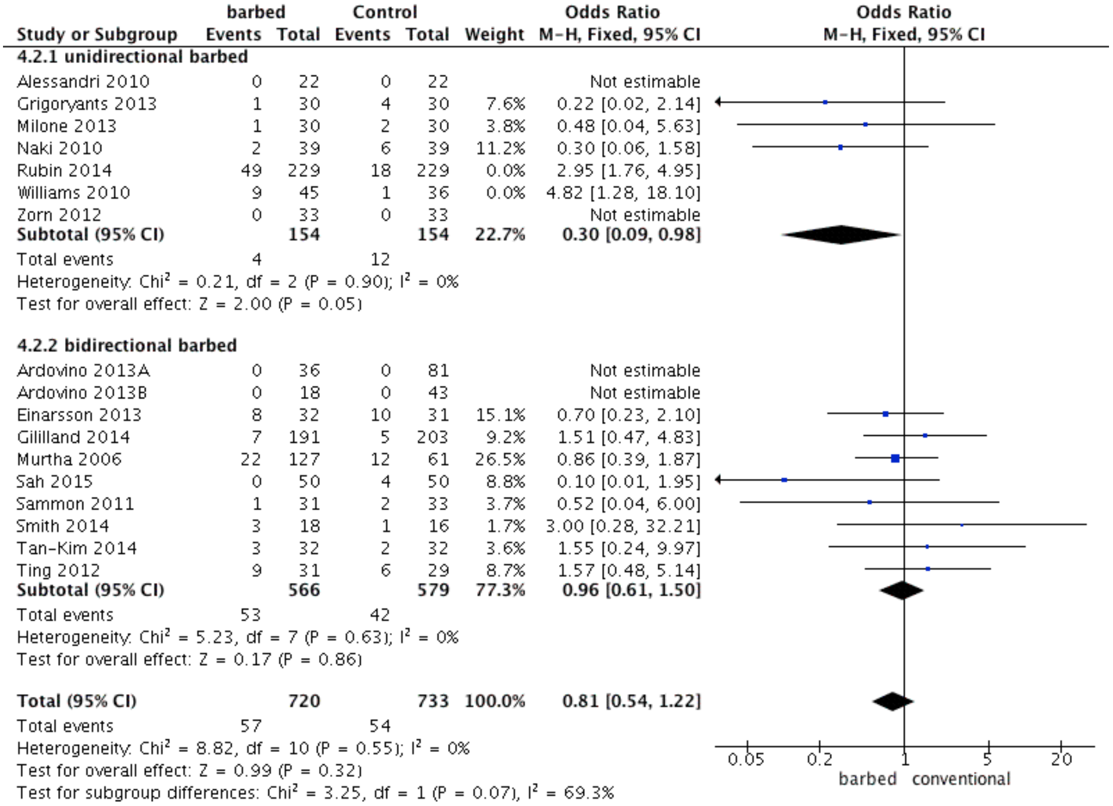

Appendix Figure 10: Funnel plot of suturing time in all included studies

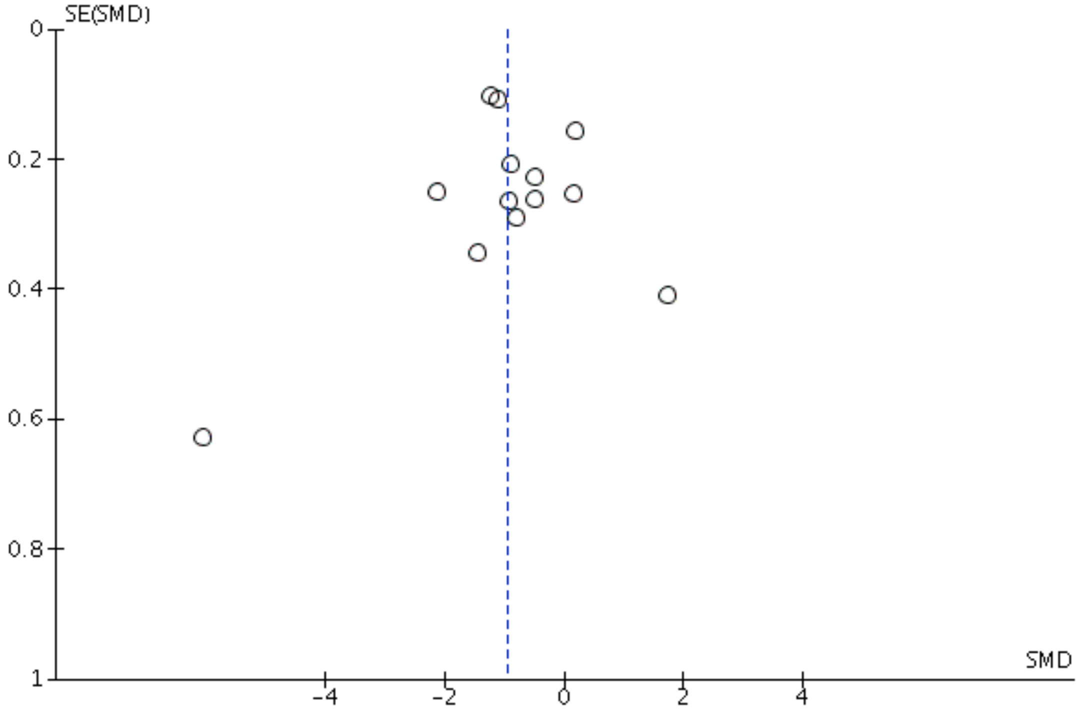

Appendix Figure 11: Funnel plot of operative time in all included studies

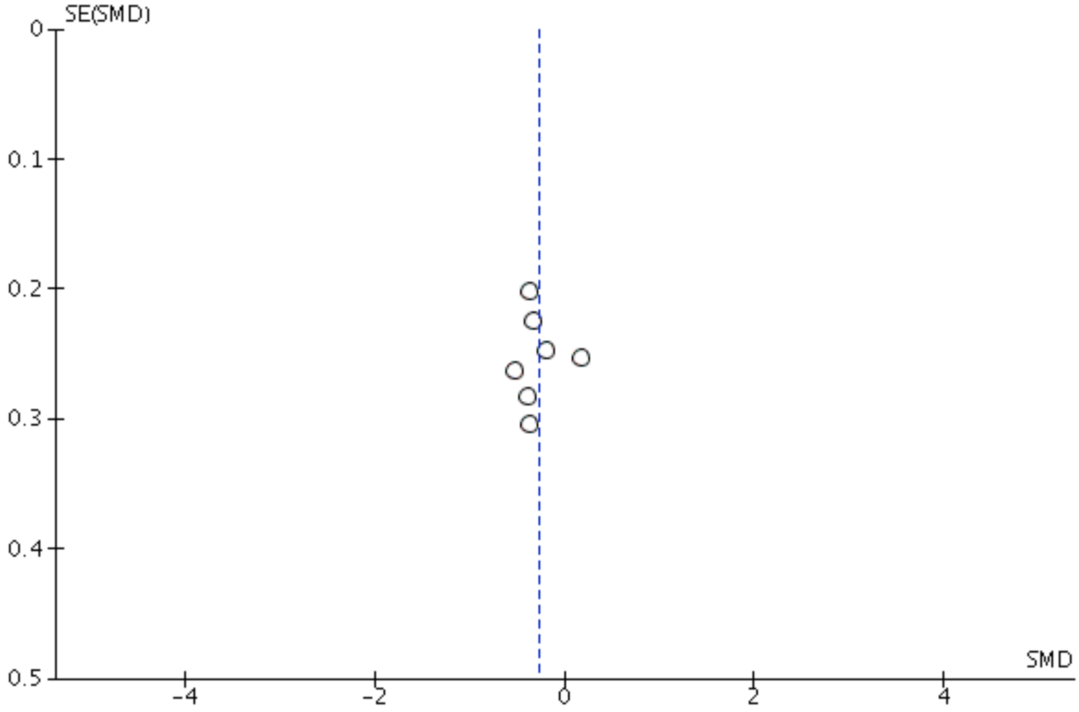

Appendix Figure 12: Funnel plot of estimate blood loss in all included studies

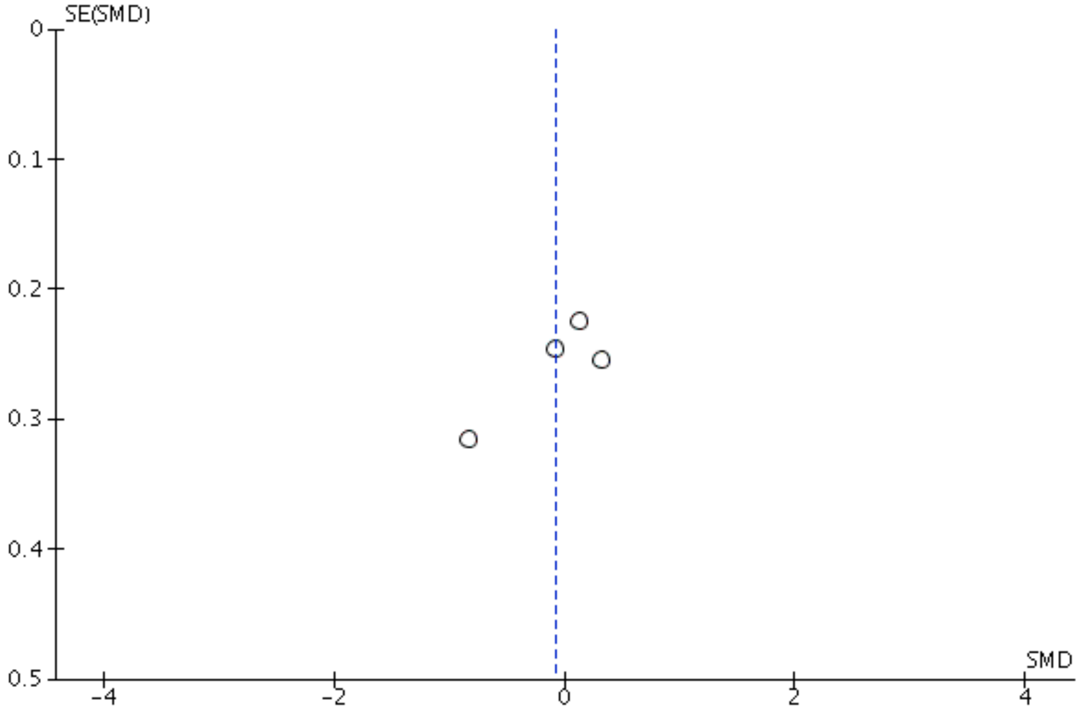

Appendix Figure 13: Funnel plot of postoperative complications in all included studies

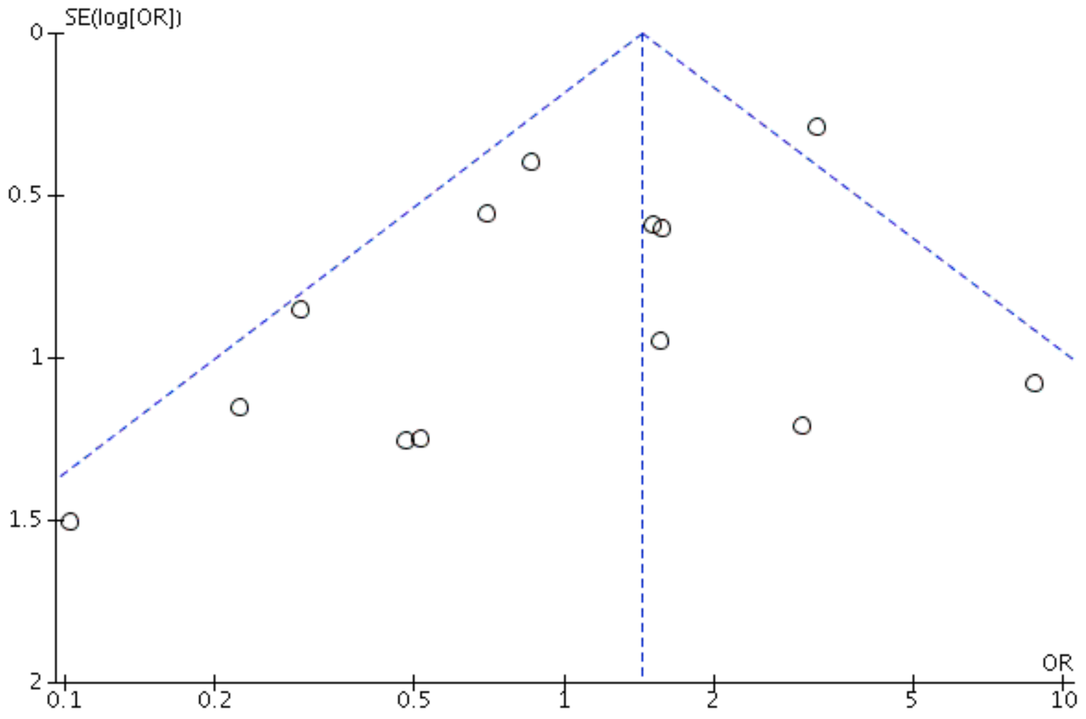
SE(log[OR])
0
0.5
1
1.5
2
0.1
0.2
0.5
1
2
5
10
OR

Appendix Table 1: Quality assessment of studies in the meta-analysis based on Cochrane recommendations

**Author year**	**Random sequence generation**	**Allocation concealment**	**Blinding of participants and personnel**	**Blinding of outcome assessment**	**Incomplete outcome data**	**Selective reporting**
Murtha 2006	Subjects were randomized within strata in the ratio of two barbed suture subjects for every one control subject	Using a closed envelope system	Subjects, but not the operating surgeon/investigators, were blinded	Primary endpoint assessment was blinded	Lost follow-up in barbed/control group N(%)：4(3.1) / 2(3.2)	Not stated
Alessandri 2010	Computer generated	Sealed opaque envelope	Not stated	Not stated	No	Not stated
Naki 2010	Computer generated	Sealed opaque envelopes	Neither subjects nor the operating surgeon/ investigators were blinded	No	No	No
Williams 2010	Computer generated	Not stated	Not stated	Not stated	Lost follow-up in barbed/control group N(%)：1(2.2)/0	Not stated
Sammon 2011	Computer generated	Not stated	Not stated	Not stated	No	Not stated
Ting 2012	Computer generated	Sealed opaque envelopes	Patients	No	No	No
Zorn 2012	After specimen entrapment, patients were randomized to either double-armed monofilament or barbed suture for PR and VUA	Not stated	Not stated	Not stated	No	Not stated
Ardovino 2013	Computer generated	Not stated	Not stated	Not stated	No	Not stated
Ardovino 2013	Computer generated	Not stated	Not stated	Not stated	No	Not stated
Einarsson 2013	Computer generated	Opaque sealed envelopes	Not stated	Not stated	No	No
Grigoryants 2013	Coin toss	Not stated	Not stated	A blinded evaluator	No	Not stated
Milone 2013	Computer generated	Sealed envelopes	Not stated	Not stated	No	Not stated

Gililland 2014	The randomization occurred in the operating room	Not stated	All patients were blinded	Not stated	No	Not stated
Rubin 2014	Computer generated	Not stated	Patients	Evaluated by an independent blinded plastic surgeon.	Lost follow-up in barbed/control group N(%)：21(18.2)/4(3.5)	No
Smith 2014	A random envelope was drawn which dictated the type of suture to be used	Sealed envelopes	Blinding the patients to the type of suture they received.	Not stated	No	Not stated
Tan-Kim 2014	Computer generated	The sealed envelope	Not stated	Blinded examiner	No	No
Sah 2015	Computer generated	Not stated	Not stated	Blind evaluation.	No	Not stated
